# The Elastic Ligature in Fistula in Ano

**Published:** 1880-11-20

**Authors:** 


### THE ELASTIC LIGATURE IN FISTULA IN ANO.

Although the use of the knife in these diseases must ever remain the most direct and preferable operation on the part of the surgeon, yet there are many patients who carry these fistulas for years, and undergo the acutest suffering several times each year, from a fear either of the operation itself, or because, from their circumstances, they are unable to remain in bed a few days after the operation. For this class of cases the elastic ligature is admirably suited, and I only wonder that as long as it has been known it is so little used. I have asked a number of my confreres, and I have not found one who had ever used it. I have operated several times by the elastic ligature, and have always found that it causes no pain, and no detention whatever from the patient's occupation. Solid rubber cord ⅛ in. in diameter (elastic ligature cord) was used, and was pulled with a force of 4 oz. The cord cuts as far as it will in about four days, and then must be tightened again. One tightening has usually been enough with me. In one case the man had suffered with a fistula many years, but always dreaded to have it cut; its external opening was ¾ in. from anus, and the internal opening 1½ in. above. This cut through in six days. The ligature was tightened on the third day. The cut produced by the ligature healed entirely in three weeks.

Another case had an old fistula never operated on. It was an inch from the anus, and entered the rectum two inches above. This was divided by the ligature in seven days, with one tightening on the fourth day. In neither case was there any inconvenience, beyond slight smarting the first day the ligature was tightened. To perform the operatian, a flexible silver probe, with an eye in one end, armed with silk, is passed through the fistula into the rectum and out through the anus. By means of a silk, the elastic cord is pulled through, the ends tightened by a pull which would raise four ounces, and they are then tied together close to the skin with silk. It is not well to have too much tension on the ligature, as it causes pain. If the ligature divides the tissues slowly, they will heal up behind it. One important point in the after-treatment (as well as in incised fistula) is to cause soft motions by Freidrichshall or Hunjadi Janos water, or a saline cathartic taken in the morning before breakfast; and immediately after the evacuation an enema of warm water, or infusion of mallow root or linseed. This puts the parts on the very best conditions for healing. For want of such treatment, one often sees the cuts for fistula remain unhealed an indefinite time.—*Western Lancet.*

